# A Cross-Cultural Comparison of an Extended Planned Risk Information Seeking Model on Mental Health Among College Students: Cross-Sectional Study

**DOI:** 10.2196/15817

**Published:** 2020-05-11

**Authors:** Zhaomeng Niu, Jessica Fitts Willoughby, Jing Mei, Shaochun Li, Pengwei Hu

**Affiliations:** 1 Rutgers Cancer Institute of New Jersey New Brunswick, NJ United States; 2 Washington State University Pullman, WA United States; 3 AI for Healthcare IBM Research Beijing China

**Keywords:** information seeking behavior, mental health, cross-cultural comparison

## Abstract

**Background:**

Approximately 42.5 million adults have been affected by mental illness in the United States in 2013, and 173 million people have been affected by a diagnosable psychiatric disorder in China. An increasing number of people tend to seek health information on the Web, and it is important to understand the factors associated with individuals’ mental health information seeking. Identifying factors associated with mental health information seeking may influence the disease progression of potential patients. The planned risk information seeking model (PRISM) was developed in 2010 by integrating multiple information seeking models including the theory of planned behavior. Few studies have replicated PRISM outside the United States and no previous study has examined mental health as a personal risk in different cultures.

**Objective:**

This study aimed to test the planned risk information seeking model (PRISM) in China and the United States with a chronic disease, mental illness, and two additional factors, ie, media use and cultural identity, among college students.

**Methods:**

Data were collected in both countries using the same online survey through a survey management program (Qualtrics). In China, college instructors distributed the survey link among university students, and it was also posted on a leading social media site called Sina Weibo. In the United States, the data were collected in a college-wide survey pool in a large Northwestern university.

**Results:**

The final sample size was 235 for the Chinese sample and 241 for the US sample. Media use was significantly associated with mental health information–seeking intentions in the Chinese sample (*P*<.001), and cultural identity was significantly associated with intentions in both samples (China: *P*=.02; United States: *P*<.001). The extended PRISM had a better model fit than the original PRISM.

**Conclusions:**

Cultural identity and media use should be considered when evaluating the process of mental health information seeking or when designing interventions to address mental health information seeking.

## Introduction

### Background

In the United States, about 42.5 million adults have been affected by various kinds of mental illness in 2013, which means that 1 in 5 Americans have had a diagnosable mental disorder [1]. In China, approximately 173 million people experience a diagnosable psychiatric disorder [2]. People in the United States who are not mental health professionals tend to recognize serious mental issues, such as schizophrenia, as mental illness. Mental disorders, such as depression and paranoia, which also require proper treatment, are often not perceived as mental disorders [3,4]. The situation is worse in China; the majority of people have a negative attitude toward mental health counseling services [5]. Some Asians see admitting to having a mental illness as bringing disgrace to the family and believe that willpower could maintain mental health to some extent [6,7]. Traditional Chinese culture and the history of medicine do not define mental illness separately from neurological disorders [8], whereas in the United Sates, psychiatrists are trying to incorporate medical treatment with counseling services that can help analyze the social and environmental factors of patients.

Moreover, Chinese culture has been known as a collectivistic culture, where group members traditionally put group interests and community values ahead of individual interests [9]. Therefore, people who suffer from mental illness may refuse to seek counseling services because of the concern that they may bring shame to the family or community. In the United States as well, there is stigma around mental health. Even among professional health care workers, negative attitudes toward mental illness are pervasive [10]. Therefore, individuals may avoid interpersonal communication and turn to the internet for information related to mental health.

With the ubiquitous use of the internet (eg, used by more than 3 billion people throughout the world) [11], people now have unparalleled access to information. In the United States, 72.00% of internet users have sought health information online in 2013[12]. In China, research has found that more than 80% of individuals experiencing a specific health condition (ie, people with epilepsy) have sought information related to their illness [13]. One study found that 18% of adult internet users in the United States had searched for mental health information online [14]. Using the internet for mental health information has been found to be prevalent among young adults. Horgan and Sweeney [15] found that 30.8% of their participants, who were college students, had used the internet for information related to mental health, conducting searches mainly on depression. This may be tied to the age of onset for mental illness; three-quarters of all chronic mental illnesses begins by the age of 24 years [16]. College may also be a risky time for students’ mental health, and college students tend to feel depressed and anxious during their college life [17]. Previous studies have found that more than 20% of college students reported mental health problems in both China [18] and the United State [19], and they are also heavy media users [20]. Thus, mental health information seeking is of importance among a college population. Therefore, a college student sample was used for this study. As the United States and China are two typical but distinct cultures that can represent different populations in mental health information seeking, a cross-cultural study was conducted in these 2 countries among college students.

### The Planned Risk Information Seeking Model

There has been a fair amount of studies about health-related information seeking since the 1990s [21]. In 2010, Kahlor [22] developed the planned risk information seeking model (PRISM) by integrating multiple information seeking models including the theory of planned behavior [23], the risk information seeking and processing (RISP) model [24], and the augmented RISP model [25]. PRISM [22] proposes that subjective norms, attitude toward seeking, and perceived seeking control will be positively associated with seeking intent; affective response to risk and perceived knowledge insufficiency will be positively related with seeking intent; perceived knowledge partially mediates the effects of attitude, seeking-related subjective norms, and perceived control on perceived knowledge insufficiency; and risk perception will be positively related to affective risk response.

When proposing PRISM, Kahlor [22] assessed the topic of general health risk information seeking, finding the model a good fit for the data, although the predicted path of knowledge insufficiency to seeking intent was not supported. Additional research has found support for PRISM to be an acceptable model for predicting information seeking intentions [26-28]. Hovick et al [29] found that original variables in PRISM better fit their data than an expanded model with past seeking behavior, source beliefs, and outcome expectancies regarding the context of cancer risk. However, Ho et al [27] extended PRISM in Singapore by adding media use and found the extended PRISM had a better fit than the PRISM. Few studies have replicated PRISM outside the United States and/or compared the relationships within PRISM under different cultural contexts. Furthermore, Willoughby and Myrick [28] found, in an examination of PRISM in two health contexts, that although some of the paths were not supported in the model, PRISM was a good fit for two health contexts: sexual health and cancer. No previous study has examined mental health as a personal risk in different cultures. This study aimed to examine whether PRISM is a good fit for the data in both China and the United States regarding mental health.

### Media Use

Although PRISM incorporates a number of constructs from various theories, it does not include the construct of media use. Media use can serve as a source of knowledge and provide important information; it can also be affected by knowledge needs and remind information seekers to stay alert. Previous research found media use to be a mediator in PRISM for predicting impersonal risk information seeking, specifically examining climate change [27]. Media use has not been assessed as a predictor in PRISM when examining personal risks. Personal risks may be more emotional than impersonal risks [30]. This study used a personal risk, mental health, as a context to replicate and extend the current model.

Demographics, ideology, personality traits, and social persuasive effects influence media use [31]. According to the reinforcing spirals framework [31], media can influence attitudes and behaviors; furthermore, media use can also influence future media use or avoidance. In information seeking, media are often seen as information sources. Media use has been found to be associated with health information seeking for impersonal risk [27,32]. In addition, people who engage with one media channel for health information are more likely to engage with other media channels in their search for health information [33].

The reinforcing spirals framework highlights the potential timeline and mechanisms for how media use might work. For example, a person who has had a family member experience depression might be primed to have certain attitudes and opinions. After seeing a commercial for a medication for depression, the person might be more likely to seek out additional information on signs and symptoms because of their own personal past experiences and also the information they were exposed to through the media.

A number of studies have shown that media use could influence perceived knowledge and intentions within the context of health [34-36]. Little research has examined the relationship between media use and variables within PRISM under the context of mental health.

### Cultural Identity

Another factor not included in PRISM is cultural identity, which is identification with a particular cultural group [37]. Even with the same cultural background, people can have different levels of cultural identity. Few studies about information seeking have examined the role of cultural values and how different levels of cultural identity affect the process of information seeking. One cross-cultural study discovered that people from different cultures, such as a communitarian culture (eg, Chinese culture) and an individualistic culture (eg, US culture), tend to perceive variables differently in risk information seeking [38]. Although this research assessed risk information seeking across cultures, it did not include cultural identify as a predictor. Our study assesses the potential role of cultural identity as a predictor of mental health information seeking.

Hofstede [39] defines culture as “the collective programming of the mind, which distinguishes one group or category of people from another.” Even under the same cultural context, individuals can experience different levels of cultural identity. Cultural identity was defined as an individual’s identification with a particular cultural group [37]. A strong cultural identity is accompanied with a deep understanding of group values, social norms, and certain behaviors endorsed by the community members [37]. For example, Chinese culture values tend to stigmatize mental health and not talk about it. If someone instead believed it was normal to have mental issues and believed it was healthy to talk with a therapist, this person’s cultural identity of Chinese culture regarding mental health would be low.

Chinese culture, as a typical Asian culture, is known as highly collectivistic with value placed on group members’ opinions and a focus on cooperation in group settings [39,40]. There is evidence that Chinese value social norms more and are more likely to maintain the existing social structure than people from individualistic cultures [41,42]. Therefore, we believe that the seeking intent of mental health information will be weaker among a Chinese sample not only because of the previous treatment of mental health in China but also because many individuals from a collectivistic culture see having mental health issues as losing face or bringing damage to the family’s reputation [6]. Moreover, the cultural value of the United States is recognized as individualistic [39], and people from individualistic cultures are more likely to accept mental health counseling [6].

### Research Questions and Hypotheses

On the basis of the previously discussed literature, we proposed the following research questions and hypotheses to help assess how the additional variables may impact behavioral intentions and the utility of the PRISM model at predicting behavioral intentions in different samples:

- Research question (RQ) 1: Is PRISM a good fit for both the Chinese sample and the US sample of young adults on the topic of mental health information seeking?
- Hypothesis 1: Media use will be positively related to seeking intention in the Chinese (H1a) and US (H1b) samples.
- Hypothesis 2: Cultural identity will be negatively associated with mental health information seeking intentions among participants in the Chinese sample.
- Hypothesis 3: Cultural identity will be positively associated with mental health information seeking intentions among participants in the US sample.
- RQ2: What are the relationships between media use and perceived knowledge, perceived knowledge insufficiency, affective response, risk perception, attitude, and subjective norms in the PRISM in the mental health information seeking process in both samples?
- RQ3: What are the relationships between cultural identity and variables of PRISM in the mental health information seeking process in both samples?
- RQ4: Does the extended PRISM account for more variance in seeking intentions than the PRISM in both the Chinese (RQ4a) and US (RQ4b) samples?

## Methods

### Sample and Procedure

Data were collected in both countries using the same online survey through a survey management program (Qualtrics). The studies were similar in all aspects, except during the recruitment phase. Participants in the United States were recruited from a college participation system called Sona, a cloud-based subject pool. Participants in China were recruited from 2 universities in China and a social media site. Although the methods differ, both provided a sample of college students that was obtained as a convenience sample. These differences in recruitment were due, in part, to logistical issues. The researchers did not have access to a participant pool in China but wanted a comparable population of college students in the same age range. The University Institutional Review Board reviewed the study proposal, and the project was determined to be exempt for both samples.

In China, 2 college instructors in 2 different universities distributed the anonymous survey link to their students using online class announcements and the survey link was also posted on a leading social media site called Sina Weibo to ensure an equivalent sample size. Sina Weibo is often seen as the “Chinese Twitter” and is a microblogging website in China. The questionnaire was translated into Chinese and was cross-examined by an English instructor of a university in China to verify whether the 2 survey versions were consistent. Two students volunteered to pretest the survey, and the completion time was around 15 min. Respondents needed to be 18 years or older to participate. One researcher posted the study description including study information, target population, survey link, and incentive information on Sina Weibo, and snowball sampling was used to distribute the survey link. At the beginning of the online survey, students needed to give their consent to participate in the study by selecting “I am 18 or older and agree to participate.” An incentive of CNY ¥260 (US $37.02) or an equivalent prize was provided in the form of a random drawing. At the end of the survey, participants were provided with another link to enter their email address for a chance to win the incentive. The email addresses were only used for the draw, and participants could not be identified by either the researchers or the instructors. The data collection lasted for about a month, and the completion rate was 77.6%. Incomplete questionnaires and surveys that took less than 2 min to complete were not included in the final analysis.

In the United States, the data were collected in a college-wide subject pool (Sona) in a large Northwestern university. Respondents were recruited among undergraduate students from different majors, and participants were offered extra credit for participating in the study. Participants signed up in Sona and then were directed to the survey in Qualtrics. Participants needed to give consent before they could proceed to the main questionnaire. The data collection process lasted less than 2 months. The completion rate was 96.78%. Students had options for alternate assignments to receive similar credit for their courses. Questionnaires that were incomplete and took less than 2 min to finish were not included in the final analysis.

### Measurement

We used the existing measures of attitude toward seeking, subjective norms, perceived seeking control, perceived current knowledge, risk perception, affective response, perceived knowledge insufficiency, and seeking intent from Kahlor [22].

#### Attitude Toward Seeking

Seven 7-point scale statements were used to measure respondents’ attitude toward seeking mental health information. Questions asked whether seeking mental health–related information was “bad” or “good,” “unhelpful” or “helpful,” “worthless” or “valuable,” “unproductive” or “productive,” “harmful” or “beneficial,” “foolish” or “wise,” and “not useful” or “useful.” Items were averaged to create a scale (United States: alpha=.95, mean 5.91, SD 1.11; China: alpha=.90, mean 5.56, SD 0.97).

#### Seeking-Related Subjective Norms

Five 5-point Likert-type items measured the degree of agreement with statements regarding subjective norms (eg, “Most people who are important to me think that I should seek information about risks to my mental health.”) Items were averaged to create a scale (United States: alpha=.92, mean 2.81, SD 1.13; China: alpha=.91, mean 2.62, SD 1.03).

#### Perceived Seeking Control

Four 5-point Likert-type items measured the degree of agreement with statements regarding perceived seeking control (eg, “I can readily access all the information about risks to my mental health that I need”). Items were averaged to create a scale (United States: alpha=.90, mean 3.47, SD 0.91; China: alpha=.91, mean 3.23, SD 0.98).

#### Risk Perception

Three 11-point items measured risk perception related to mental health (0=not at all and 10=extremely) with the following statements: “How serious are the current threats to your mental health?” “How likely are you to have some mental health issues in the next year?” and “If you were to have some mental health issues in the next year, how serious do you think it would be?” Items were averaged to create a scale (United States: alpha=.88, mean 3.86, SD 2.31; China: alpha=.88, mean 5.32, SD 2.30).

#### Affective Response

Two 5-point Likert-type items asked respondents to indicate their degree of worry and fear. The statements were “Current risks to my mental health are scary” and “Current risks to my mental health are worrisome.” The items were averaged to create a scale (United States: alpha=.94, mean 2.39, SD 1.26; China: alpha=.89, mean 2.76, SD 1.14).

#### Perceived Current Knowledge

A statement measured perceived current knowledge by asking respondents the following: “Rate your mental health risk knowledge on a scale of 0 to 100, where zero means knowing nothing about risks to your mental health and 100 means knowing everything you could possibly know about risks to your mental health” (United States: mean 57.48, SD 24.30; China: mean 64.09, SD 18.06).

#### Perceived Knowledge Insufficiency

The measurement of sufficiency threshold asked the following: “Think of that same 0 to 100 scale again. This time, estimate how much knowledge you need to deal adequately with risks to your mental health. You might feel you need the same, more, or possibly even less information about this topic. Using a scale of zero to 100, how much information would be sufficient for you” (United States: mean 66.14, SD 23.45; China: mean 74.92, SD 17.34). 

#### Seeking Intentions

Five 5-point Likert-type items measured seeking intent (eg, “I plan to seek more information about risks to my mental health in the near future”). Items were averaged to create a scale (United States: alpha=.97, mean 2.90, SD 1.07; China: alpha=.94, mean 2.98, SD 1.01).

#### Media Use

We adapted Brossard and Nisbet’s [43] scale. Eight 7-point items measured participants’ attention paid to mental health information in health magazines, newspapers, TV news reports, entertainment TV programs, online forums, social media, news reports on the internet, and medical applications (eg, “How much attention have you paid to mental health information on social media?”). Items were averaged to create a scale (United States: alpha=.82, mean 3.34, SD 1.17; China: alpha=.84, mean 3.47, SD 1.22).

#### Cultural Identity

We measured cultural identity with Usborne and Taylor’s [37] Cultural Identity Clarity Scale. Eight 11-point items measured respondents’ agreement or disagreement with statements such as “My beliefs about my cultural group often conflict with one another” and “My beliefs about my cultural group seem to change very frequently.” Some items were reverse coded, and all items were averaged to create a scale (United States: alpha=.87, mean 5.68, SD 1.89; China: alpha=.76, mean 5.33, SD 1.67).

### Data Analysis

As we wanted to examine a college student sample across both countries, we only used those who were college students in the both samples. As the measurements were used in former studies and have high reliability (high Cronbach alpha), we computed the items to create one variable for each construct and then conducted path analyses in Mplus version 7.11 to evaluate the paths and model fit. The models included gender as a control variable.

## Results

### Sample Statistics

After data cleaning, each sample had more than 200 participants complete the survey (United States: N=241; China: N=235). In the US sample, more than one-third of the sample reported being male (n=83) and less than three-quarters, female (n=158). Participants’ ages ranged from 18 to 32 years (mean 20 years, SD 1.97). Almost two-thirds of the respondents (n=160) reported being white and >10.0% (n=27) as Asian. In the Chinese sample, almost 90.0% (n=209) were female. Respondents ranged from 18 to 27 years (mean 21 years, SD 7.98). Basic descriptive results of the variables are shown in Table 1.

**Table 1:** Basic descriptive results.

Variable	United States, mean (SD)	China, mean (SD)	Range
Attitude toward seeking	5.91 (1.11)	5.56 (0.97)	1-7
Seeking-related subjective norms	2.81 (1.13)	2.62 (1.03)	1-5
Perceived seeking control	3.47 (0.91)	3.23 (0.98)	1-5
Risk perception	3.86 (2.31)	5.32 (2.30)	0-10
Affective response	2.39 (1.26)	2.76 (1.14)	1-5
Perceived current knowledge	57.48 (24.30)	64.09 (18.06)	0-100
Perceived knowledge insufficiency	66.14 (23.45)	74.92 (17.34)	0-100
Seeking intentions	2.90 (1.07)	2.98 (1.01)	1-5
Media use	3.34 (1.17)	3.47 (1.22)	1-7
Cultural identity	5.68 (1.89)	5.33 (1.67)	0-10

### Model Fit

For the Chinese sample, the replicated PRISM did not have a good fit, whereas the extended model with 2 additional variables, attention to media and cultural identity, had a good model fit. The replicated PRISM of the US participants did not have a good model fit, whereas the extended PRISM of the US sample had an acceptable model fit (see Table 2 for all model fit information). According to Browne and Cudeck [44], root mean squared error of approximation values less than 0.08 can be considered as the reasonable error of approximation. A comparative fit index and Tucker-Lewis index close to or greater than 0.95 represent a good fit. A normed chi-square value less than 5 indicates a good fit [45]. Thus, RQ1 was answered.

**Table 2:** Summary of model fit.

Model	Chi-square (*df*)	Root mean squared error of approximation	Comparative Fit Index	Tucker-Lewis Index
China PRISM^a^	55.4 (2.41)	0.078	0.91	0.89
China Extended PRISM	57.1 (1.59)	0.050	0.96	0.93
US PRISM	90.1 (3.91)	0.110	0.77	0.70
US Extended PRISM	75.4 (2.09)	0.067	0.92	0.86

^a^PRISM: Planned Risk Information Seeking Model.

### The Planned Risk Information Seeking Model of the Chinese Sample

**Figure 1:**
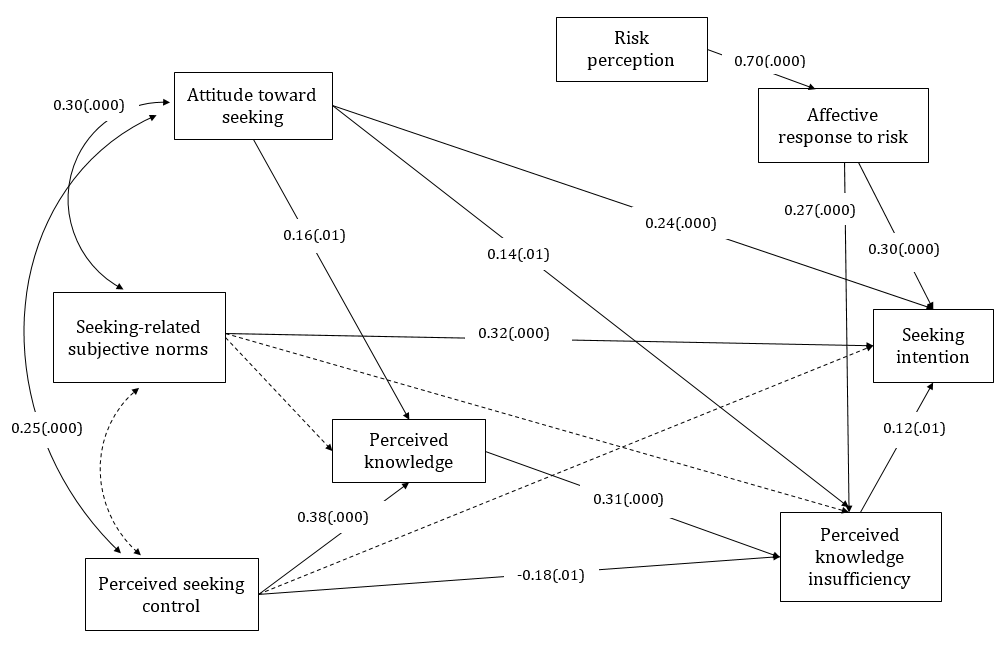
The planned risk information seeking model of the Chinese sample. Dashed lines denote hypothesized nonsignificant paths. The model includes effects of control variables, which are not displayed. (.000) represents significant path coefficients at the .001 level and (.01), at the .05 level.

Some significant paths of Kahlor’s [22] PRISM were not significant in the Chinese sample (Figure 1) Perceived seeking control was not significantly related to seeking intention, seeking-related subjective norms were not significantly related to perceived information insufficiency, and seeking-related subjective norms were not significantly related to perceived current knowledge.

### The Planned Risk Information Seeking Model of the US Sample

**Figure 2:**
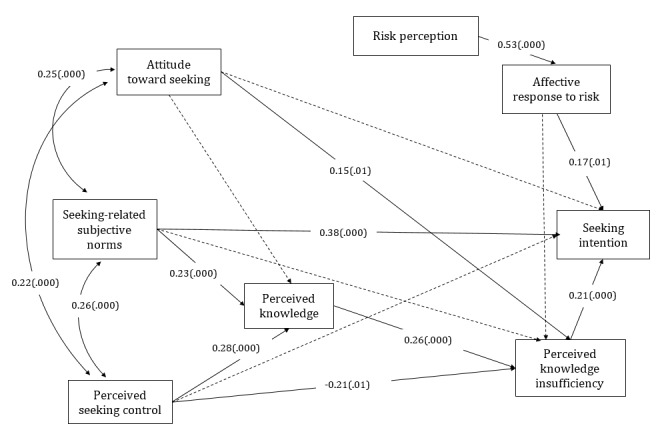
The planned risk information seeking model of the US sample. Dashed lines denote hypothesized nonsignificant paths. The model includes effects of control variables, which are not displayed. (.000) represents significant path coefficients at the .001 level and (.01), at the .05 level.

The paths and standardized coefficients of the replicated PRISM of the US sample are presented in Figure 2.

### The Extended Planned Risk Information Seeking Model of the Chinese Sample

**Figure 3:**
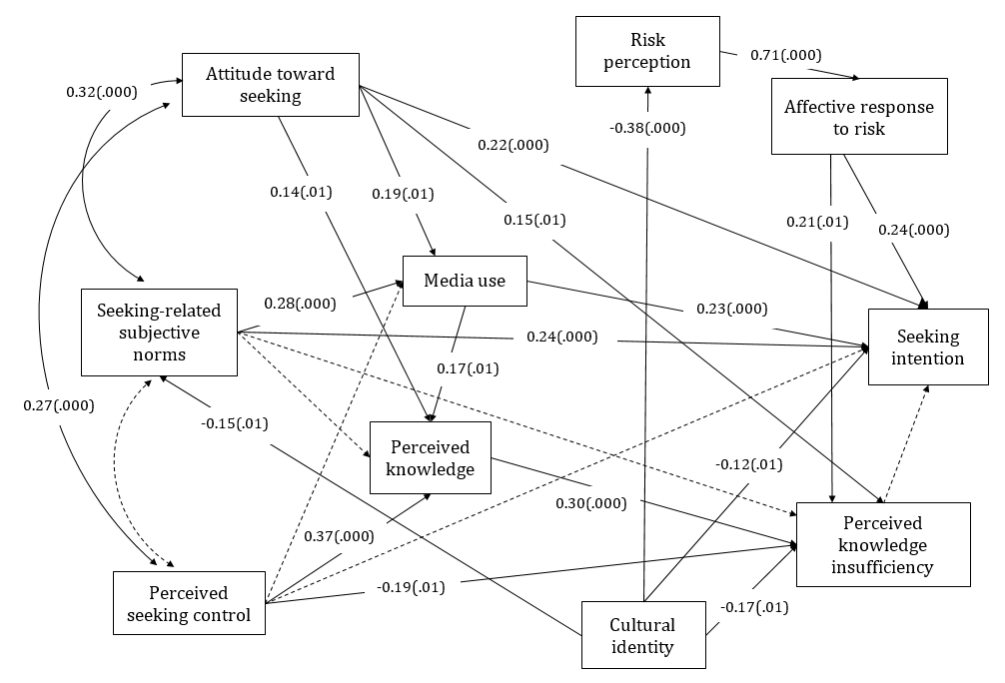
The extended planned risk information seeking model of the Chinese sample. Dashed lines denote hypothesized nonsignificant paths. The model includes effects of control variables, which are not displayed. (.000) represents significant path coefficients at the .001 level and (.01), at the .05 level.

The Chinese extended model with media use and cultural identity are shown in Figure 3. Attitude toward seeking (β=.22; *P*<.001), seeking-related subjective norms (β=.24; *P*<.001), affective response to risk (β=.24; *P*<.001), media use (β=.23; *P*<.001), and cultural identity (β=−.12; *P*=.02) were significantly associated with information seeking intention. Therefore, H1a and H2 were supported.

In addition, attitude toward seeking (β=.15; *P*=.02) and seeking-related subjective norms (β=.28; *P*<.001) and perceived knowledge (β=.17; *P*=.002) were significantly associated with media use. Cultural identity was significantly associated with perceived knowledge insufficiency (β=−.17; *P*=.003), risk perception (β=−.38; *P*<.001), and seeking-related subjective norms (β=−.15; *P*=.02). These answered RQ2 (What are the relationships between media use and the variables of the PRISM?) and RQ3 (What are the relationships between cultural identity and variables of the PRISM?) in the Chinese sample.

The replicated Chinese PRISM accounted for 35.9% of the variance in information seeking intention, whereas the extended model in the Chinese sample accounted for 41.4% of the variance in information seeking intention, answering RQ4a.

### The Extended Planned Risk Information Seeking Model of the US Sample

**Figure 4:**
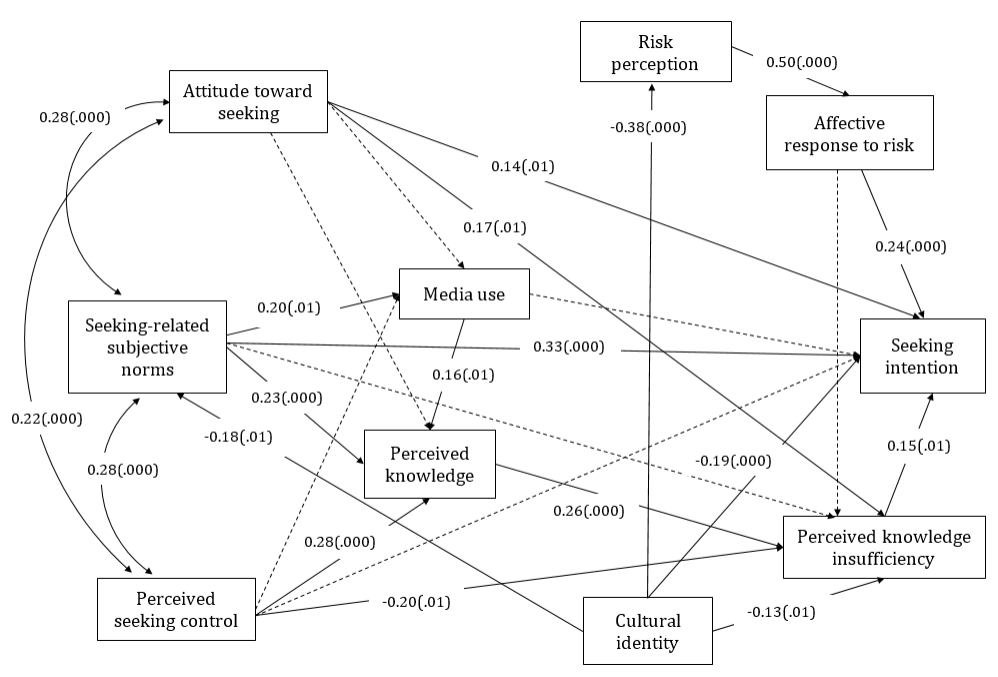
The extended planned risk information seeking model of the US sample. Dashed lines denote hypothesized nonsignificant paths. The model includes effects of control variables, which are not displayed. (.000) represents significant path coefficients at the .001 level and (.01), at the .05 level.

In the US extended PRISM (see Figure 4), attitude toward seeking (β=.14; *P*=.005), seeking-related subjective norms (β=.35; *P*<.001), perceived knowledge insufficiency (β=.14; *P*=.005), affective response to risk (β=.24; *P*<.001), and cultural identity (β=−.19; *P*<.001) were significantly associated with information-seeking intentions. Thus, H1b and H3 were not supported.

In addition, media use was significantly associated with subjective norms (β=.20; *P*=.001) and perceived knowledge (β=.16; *P*=.004). Perceived knowledge insufficiency (β=−.13; *P*=.02), risk perception (β=−.38; *P*<.001), and seeking-related subjective norms (β=−.18; *P*=.002) were significantly associated with cultural identity. These answered RQ2 (What are the relationships between media use and the variables of the PRISM?) and RQ3 (What are the relationships between cultural identity and variables of the PRISM?) in the US sample.

In the US sample, the PRISM accounted for 30.6% of the variance in information seeking intention, whereas the extended model accounted for 40.3% of the variance in information seeking intention. So RQ4b, which asked whether the extended PRISM accounted for more variance in seeking intention than the PRISM in the US sample, was answered.

## Discussion

### Principal Findings

In this study, we tested the PRISM model in a sample of US and Chinese college students in the context of mental health. We then added additional variables to the model and compared results across cultures. In terms of the model, the original PRISM model was not a great fit with our data in either samples. Consistent with the results of Kahlor [22], we found that attitude toward seeking and seeking-related subjective norms significantly predicted intention to seek mental health information in both samples. In addition, attitude toward seeking and perceived seeking control significantly predicted perceived knowledge insufficiency. Perceived seeking control influenced seeking intention indirectly by its effects on perceived knowledge and perceived knowledge insufficiency in both samples. Some studies have found a significant role of perceived control in predicting intent to seek help regarding mental health in China [46]. However, perceived control of searching for mental health information does not require professional evaluations, accessibility, cost, and time, which indicates that for personal risks, such as mental health, perceived seeking control may not directly be related to information seeking intention. Risk perception was found to have a direct effect on affective response to risk in all of our models, consistent with the path in the PRISM [22]. Affective response to risk significantly predicted seeking intention and perceived knowledge insufficiency in both samples.

The extended PRISM that we tested included 2 additional variables, ie, media use and cultural identity, and had a better model fit than the PRISM in both the US and Chinese samples. An important finding in this study is the role of media use in PRISM. Our finding is inconsistent with Ho et al’s [27] study. Their study demonstrated that media use impacted risk perception and impacted affective response directly and seeking intention indirectly regarding climate change among a Singapore population; however, among our Chinese sample, we found that media use was directly associated with intention to seek mental health information. Mental health as a personal threat could produce a stronger emotional response than impersonal risks, such as climate change [27]. People in China who pay more attention to this issue may have a greater intention to explore more about this personal risk, as they have limited knowledge of professional mental health services and counseling. Another explanation is that people who pay more attention to mental health information have the need for mental health information, so they intend to seek more information regarding mental health [47]. In the Chinese sample, media use was influenced by attitude toward seeking and subjective norms and impacted perceived knowledge, which is consistent with the findings of Ho et al [27]. In the US sample, media use was influenced by subjective norms and had influences on perceived knowledge. We did not find a direct effect of media use on seeking intention in the US sample, which is consistent with the study by Ho et al [27].

Another contribution of this study was that cultural identity significantly predicted seeking intention, perceived knowledge insufficiency, risk perception, and subjective norms related to information seeking in both samples, which contributes to the extended PRISM in predicting personal risk. However, cultural identity negatively predicted seeking intention in both samples, which indicates that lower levels of identification of the cultural group is associated with higher levels of seeking intention toward mental health information seeking. We predicted that cultural identity would have opposite effects on the Chinese and US participants’ information seeking intentions based on the assumption that mental health problems are stigmatized in China while less so in the United States. However, the results showed that people who are less identified with the cultural values regarding mental health issues in their cultures are more likely to seek mental health information, which suggests that mental health issues are stigmatized to some extent in both samples.

Cultural identity is positively related to self-esteem and well-being [48]. If the respondent scored lower on cultural identity, he or she is more likely to have an unclear group identity. This finding reveals that for an issue, which was perceived as stigmatized in both individualistic and collectivistic cultures [6,10], a lower level of cultural identity may act as an unclear group identity to reduce the constraints of a group culture, which could have a direct influence on mental health information seeking. Previous research has found that an unclear cultural identity could reflect a high level of normlessness, which means no clear norms guidance [37]. We found that lower levels of cultural identity were associated with high levels of subjective norms related to mental health information seeking, which means that perceptions of the people with a lower cultural identity about others’ expectations on mental health information seeking are inconsistent with the majority of people in the society. Several studies have shown that culture has an impact on risk perceptions among different culture groups [38,49]. On the basis of our results, people with unclear or dissimilar group identities tend to have higher risk perception about mental health information seeking.

Overall, we found that media use and cultural identity can be two variables useful for predicting seeking intentions regarding personal risks such as mental health. With 2 additional variables added in the PRISM, variance accounted for in seeking intentions by the models increased in both samples. Owing to the specificity of the context, the 2 samples with different cultural backgrounds have distinguished results in some perspectives, which again emphasize the importance of culture. We believe that the traditional thought of mental health in China played an important role in explaining these findings as mental health is still a stigmatized issue in China. In addition, the mental health counseling system is not as thoroughly developed in China [50] as in the United States, making this topic less familiar among our Chinese sample. Such differences in perceptions of mental health may directly alter the disease progression. As a chronic disease, early interventional treatment of mental health and the access to additional information during long-term treatment can significantly affect the prognosis of patients, which illustrate the importance of correctly recognizing a mental health issue at an early stage.

These findings can be used in campaigns promoting the seeking of mental health information and can help researchers and practitioners understand the process of personal risk information seeking, especially when the topic has a strong cultural context. The findings also have theoretical implications. Some of the paths are inconsistent from past research regarding personal cancer risk using PRISM [29], and we found that additional variables may provide a better fit for the models based on our topic of mental health.

### Limitations and Future Studies

This study is not without limitation. First, we used a convenience sample for both populations. The results of using the sample of college students may not generalize to other subgroups, although it does allow us to assess relationships between key constructs. Second, this study used cross-sectional data and only measured variables at a specific time point without the ability to decide causality. Third, due to a relatively small sample size, we chose to conduct a path analysis. Further studies could conduct a structural equation model. In addition, as our context is a personal risk and often a stigmatized issue, future studies could use a more general concept to examine the role of media use and cultural identity. Moreover, the spiral role of media use should be explored in the future as media use can also influence risk perception and other variables such as attitudes and subjective norms.

### Conclusions

Overall, this study extended the PRISM to include important aspects that could vary based on culture, including cultural identity and media use. We found that both cultural identity and media use were associated with information-seeking intentions regarding mental health topics and including the variables in the PRISM allowed the model to account for additional variance in information-seeking intentions. These results can help researchers and health practitioners as they continue to grapple with sensitive health and risk issues, such as mental health. Potential patients may benefit from the findings, changing the progression of the disease and how it is treated, leading to more appropriate treatment solutions.

## Abbreviations

PRISM: planned risk information seeking model
RISP: risk information seeking and processing
